# Artificial Neural Network Prediction of Retention of Amino Acids in Reversed-Phase HPLC under Application of Linear Organic Modifier Gradients and/or pH Gradients

**DOI:** 10.3390/molecules24030632

**Published:** 2019-02-11

**Authors:** Angelo Antonio D’Archivio

**Affiliations:** Dipartimento di Scienze Fisiche e Chimiche, Università degli Studi dell’Aquila, Via Vetoio, 67100 Coppito, L’Aquila, Italy; angeloantonio.darchivio@univaq.it; Tel.: +39-0862-433777

**Keywords:** amino acids, reversed-phase liquid chromatography, gradient elution, retention prediction, artificial neural network

## Abstract

A multi-layer artificial neural network (ANN) was used to model the retention behavior of 16 *o*-phthalaldehyde derivatives of amino acids in reversed-phase liquid chromatography under application of various gradient elution modes. The retention data, taken from literature, were collected in acetonitrile–water eluents under application of linear organic modifier gradients (φ gradients), pH gradients, or double pH/φ gradients. At first, retention data collected in φ gradients and pH gradients were modeled separately, while these were successively combined in one dataset and fitted simultaneously. Specific ANN-based models were generated by combining the descriptors of the gradient profiles with 16 inputs representing the amino acids and providing the retention time of these solutes as the response. Categorical “bit-string” descriptors were adopted to identify the solutes, which allowed simultaneously modeling the retention times of all 16 target amino acids. The ANN-based models tested on external gradients provided mean errors for the predicted retention times of 1.1% (φ gradients), 1.4% (pH gradients), 2.5% (combined φ and pH gradients), and 2.5% (double pH/φ gradients). The accuracy of ANN prediction was better than that previously obtained by fitting of the same data with retention models based on the solution of the fundamental equation of gradient elution.

## 1. Introduction

Reversed-phase high-performance liquid chromatography (RP-HPLC) is an extensively applied technique in the separation and determination of a wide range of multi-class compounds, including biomolecules, pharmaceuticals, and industrial chemicals, in human, environmental, and food samples [1,2,3,4]. Separation of complex mixtures by RP-HPLC generally requires the application of mobile-phase gradients to overcome the typical disadvantages of isocratic elution, such as poor resolution of early peaks, broadening of late peaks, band tailing, and long separation times [5,6]. In organic modifier mobile-phase gradients (φ gradients), the concentration of organic solvent in the mobile phase is increased, determining a progressive increase of the elution power of the eluent during the gradient run and a consequent decrease in solute retention. A similar effect occurs in the pH gradient of the mobile phase [7], where an increase or decrease in pH in the case of weak bases or acids, respectively, produces a progressive increase of the ionized form of the analyte and a consequent decrease in its retention time.

In the last few decades, various predictive models [8,9,10,11] were proposed with the aim of supporting the empirical strategies commonly utilized in the development of the chromatographic methods, which can be particularly slow and inefficient when a large number of parameters have to be fixed, such as in the case of programmed elution analysis.

Many attempts to describe the retention of solutes in RP-HPLC under the application of mobile-phase gradients are based on the solution of the fundamental equation of gradient elution [12,13,14,15,16],

(1) $$\int_{0}^{t_{R-}t_{0}}\frac{\mathrm{dt}}{t_{0}k}=1$$

where t_R_ is the retention time, t_0_ is the column hold-up time, and k is the retention factor. Analytical or numerical solutions of Equation (1) require the dependence of k upon the mobile-phase composition. To this end, popular relationships relating k and φ, or empirical models arising from the experimental properties of the system are often used, where the adjustable eluent- and sometimes solute-dependent parameters associated with these relationships are determined by appropriate fitting algorithms applied to the retention data.

Artificial neural networks (ANNs), since their introduction in 1990s, are used as regression tools to address various complex issues in chromatography. The main advantage of ANN regression is that both multilinear and non-linear phenomena can be handled without the need of prior definition of a fitting function. The ANN-based applications in retention prediction include the development of quantitative structure–retention relationships (QSSRs) [17,18], modeling of the combined effects of solute structure and separation conditions (column, eluent, or both) [19,20], and transfer of retention data between different columns or eluent types [21,22,23]. ANN models based simultaneously on molecular descriptors and instrumental conditions associated with the elution mode were used to predict the retention times of diverse sets of organic compounds in gradient RP-HPLC [24,25,26,27]. We previously used ANN regression to model the retention times of 16 selected purines, pyrimidines, and nucleosides under the application of multilinear φ gradients [28]. With this aim, a network was trained to associate the retention times with both gradient profiles and solutes, the latter being represented by “bit-string” categorical descriptors, which, unlike the aforementioned QSSR-inspired approaches, did not require any assumption of the chemical structure of the analytes. The generalization ability of the so-obtained model was tested on external multilinear gradients, providing an accurate prediction of the solute retention times (within 2–3% on average). This approach was here extended to the RP-HPLC retention of ionizable solutes of biological relevance, such as amino acids, analyzed under the application of linear φ gradients, pH gradients, or combined φ/pH gradients, whereby the target compounds were previously derivatized with *o*-phthalaldehyde (OPA) to allow their fluorescence detection. The data investigated in the present study were taken from three works of Pappa-Louisi and co-workers [14,15,16], who collected the experimental data and developed retention models based on the solution of the fundamental equation of gradient elution to verify the accuracy of the predicted retention by different equations or fitting algorithms.

The present study is aimed at exploring the capability of ANN regression calibrated with the retention data collected in representative gradients to predict the chromatographic behavior of ionizable solutes in external separation conditions. Retention in gradient RP-HPLC is governed by several factors, such as the chemical structure of solutes, their acid–base properties, the polarity/acidity of the mobile phase, and how these properties change during the chromatographic run. While, on the one hand, ANN is potentially able to treat such complexity, on the other, the network does not provide a fitting equation that could be useful for getting information about the relative role of the different factors in the retention process. Nevertheless, finding the optimal condition for the chromatographic separation of a complex mixture, which is anyway a multivariate problem, can be handled by statistical retention models, but their predictive performance is more important than the knowledge of their physical meaning. In this view, a network was trained to associate the experimental parameters describing the gradient elution profile with the retention times of a mixture of target analytes to be separated. The ANN-based model, once calibrated on a sufficiently large set of representative separation conditions, was later applied to simultaneously predict the retention times of all the solutes in external elution conditions. In the end, the ANN response can be useful for optimization purposes, because it allows deducing the retention of the target solutes at any point of the experimental domain explored in calibration, and it may replace or support inefficient trial-and-error empirical approaches usually adopted to search the optimal separation conditions. At first, two separate ANN-based retention models were generated to predict the data collected under application of linear φ gradients or pH gradients. In addition, the retention behavior of the amino acids under the independent or simultaneous application of linear φ and pH gradients was modeled by ANN. The predictive performance of the various ANN-based models developed in this work was compared with the prediction ability of the retention models based on the solution of the fundamental equation of gradient elution.

## 2. Results

### 2.1. Identification of Model Variables and Data Subsets

In this paper, ANN regression was used to model the RP-HPLC retention of *o*-phthaladehyde (OPA) derivatives of 16 amino acids collected under the application of φ gradients, pH gradients, or combined pH/φ gradients. The retention datasets (A, B, and C, respectively), taken from the literature [14,15,16], are described in Section 3.1. The following variables were considered to describe the linear φ gradients of dataset A: the starting pH (pH_i_), the starting organic solvent content (φ_i_), and the φ-gradient slope (Δφ/t_g_ = (φ_f_ − φ_i_)/t_g_), where φ_f_ is the φ value at the end of gradient run and t_g_ is the gradient time. The pH gradients of dataset B were described by φ_i_, pH_i_ and the gradient slope (ΔpH/t_g_ = (pH_f_ − pH_i_)/t_g_, where pH_f_ is the final pH value). The respective values of the above quantities, determined from the experimental conditions reported in the original papers [14,16], are collected in Table 1. Among these parameters, the constant ones (ΔpH/t_g_ and φ_i_ in dataset A, and Δφ/t_g_ in dataset B) were not considered as network inputs in ANN modeling of φ gradients or pH gradients. Datasets A and B were successively fused in one comprehensive dataset, hereafter indicated as A+B, to attempt ANN modeling of retention data collected under independent applications of φ gradients and pH gradients. In this case, all four gradient descriptors reported in Table 1 are informative and were considered as ANN inputs. To describe the 27 double pH/φ gradients of dataset C (referring to double pH/φ gradients), the three non-constant experimental quantities (pH_f_, φ_f_, and t_g_) varying according to a three-level experimental design in Reference [15] were assumed as ANN inputs. The level values selected for these variables are given in Section 3.1.

**Figure 1:**
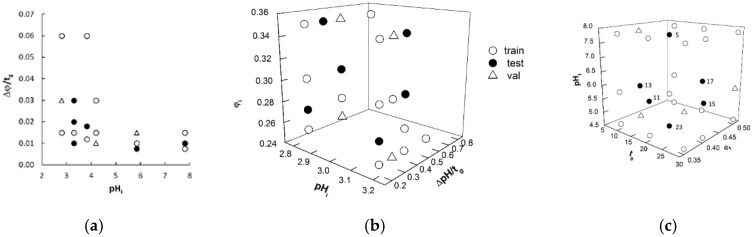
Gradients used in artificial neural network (ANN) training, validation, and test data projected in the space of the variables adopted as network inputs for datasets A (**a**), B (**b**), and C (**c**). Test samples of dataset C are labeled according to Reference [15].

As described in Section 3.2, ANN regression requires a training set, which is processed to update the network weights and biases; however, the network performance must also be monitored during learning using unknown data (validation set) to avoid overfitting. Moreover, the real generalization ability of the learned network must be finally evaluated on external data (test set) neither used in training nor in validation. To design these three datasets, the various φ gradients of dataset A, the pH gradients of dataset B, and the φ/pH gradients of dataset C were graphically represented in the space of the variables previously selected to describe the changes in the eluent composition (Figure 1). These plots helped us generate three well-balanced subsets in terms of representativeness; the data samples assigned to each subset were selected to cover the investigated experimental domain as much as homogeneously possible. Regardless of the dataset, six gradients were selected for the final test; three gradients (dataset A) or four gradients (datasets B and C) were selected for the internal validation and the remaining elution conditions were used to train the networks (Table 1 and Figure 1). The training, validation, and test sets for dataset A+B were designed by fusing the respective subsets of the A and B matrices. Considering that the retention data of 16 amino acids are associated with each experimental elution mode, the training, validation, and test data points were 160, 48, and 96, respectively, for dataset A; 192, 64, and 96, respectively, for dataset B; 352, 112, and 196, respectively, for dataset A+B; and 272, 64, and 96 for dataset C. Rather than representing the solutes by molecular descriptors, according to conventional QSRR approach, each of the 16 amino acids was identified by a 16-bit string, consisting of all “0” values except the *n*-th bit, which was set to “1”, where *n* corresponds to the position of that solute in an arbitrary and predefined sequence of the investigated analytes. In this condition, the network was trained to properly associate the retention times to both solutes and gradient modes, without any explicit reference to the solute molecular structure.

### 2.2. ANN Modeling of Retention

The distinct networks handling the retention datasets A, B, A + B, and C were optimized following a usual procedure aimed at founding the combination of the ANN adjustable parameters providing the lowest validation error. A range-scaling between 0 and 1 was always applied to both input and output variables. Retention time (t_R_(min)) values and their logarithmic values were alternatively considered as the ANN responses. Both options provided good ANN models and a random distribution of absolute residuals; however, logarithmic transformation of retention times was preferred to the unscaled values because it gave lower relative errors for the less retained amino acids.

Based on the results of preliminary ANN runs, in which a sigmoid or a tangent hyperbolic activation function was applied to the hidden neurons, the latter was preferred, while application of a non-linear transformation in the output neuron was not required because it did not produce any improvement in the model performance. The number of hidden neurons was varied in the range between *N* − 6 and *N* + 6, where *N* is the number of inputs, and each tested network was trained until the validation error reached a minimum value.

**Figure 2:**
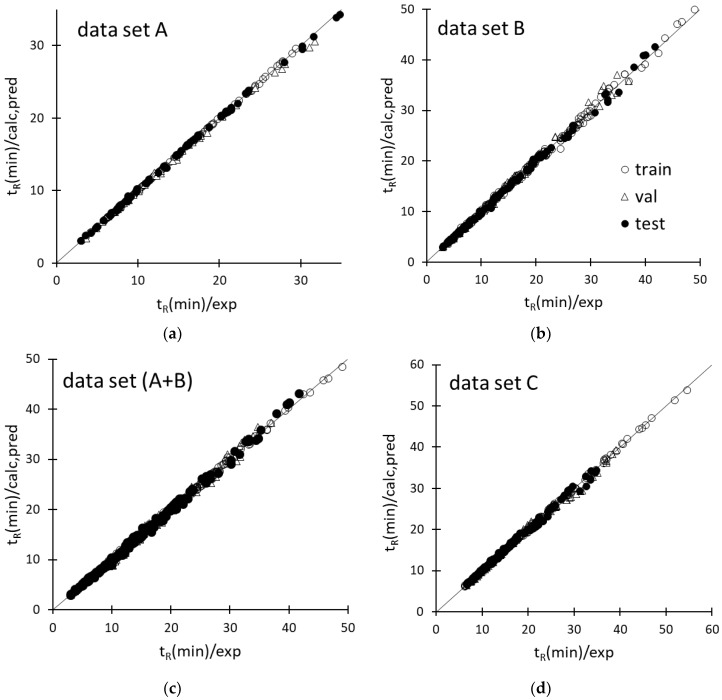
Agreement between the experimental retention times (t_R_(min)/exp) of solutes and calculated or predicted ANN responses (t_R_(min)/calc,pred) of datasets A (**a**), B (**b**), A+B (**c**), and D (**d**).

The best ANN architectures and learning durations are presented in Table 2. Because of a randomization of the starting weights, here generated between −0.1 and 0.1, the optimal network produced slightly different responses upon being re-trained several times. To minimize the influence of the initial weights on the ANN-based model performance, the network was re-trained 100 times and the outputs were averaged. The agreement between computed or predicted ANN responses and the experimental t_R_ values for each retention dataset are graphically shown in Figure 2. Table 2 displays the determination coefficients in calibration and prediction (*R*^2^ and *Q*^2^) and the related standard errors (SEC and SEP, respectively) associated with the ANN-based models, where *Q*^2^ was determined according to Todeschini et al. [29]. The average and maximum absolute percentage errors (mean(%) and max(%), respectively) in each subset are also reported. All the above statistical parameters refer to the unscaled t_R_ values.

### 2.3. Predictive Performance of the ANN-Based Models

Inspection of the agreement plots for the various retention datasets modeled by ANN (Figure 2) reveals that both computed and predicted responses were very close to the ideal line, ensuring an accurate prediction of the retention times of the amino acids within the respective experimental domains. As expected, the training data samples were better modeled than the validation and test data; nonetheless, worsening of the predictive performance as compared to the fitting ability was slight, as confirmed by the small differences among the statistical parameters of training, validation, and test sets, reported in Table 2. The data samples were also randomly distributed around the ideal line of the agreement plots, suggesting the absence of systematic errors, except for dataset C, for which a small group of validation cases in the t_R_ range between 30 and 40 min were all underestimated (Figure 2d). Most of these data samples were associated with the most retained amino acids analyzed under the application of a same gradient (φ_f_ = 0.5, pH_f_ = 5.86, t_g_ = 30 min), but the errors were anyway acceptable (within 4–7%). The retention data collected under the application of φ gradients and pH gradients were very well modeled according to the mean errors, which were smaller than 1% and 1.5% for the training and test data, respectively (Table 2). Only a slight worsening of the descriptive/predictive ANN performance was observed when the network was called to model the retention times of the amino acids under the independent application of φ gradients and pH gradients (dataset A+B) or double pH/φ gradients; the mean error in both cases was just above 1% for the training set and 2.5% for the test set (Table 2).

**Figure 3:**
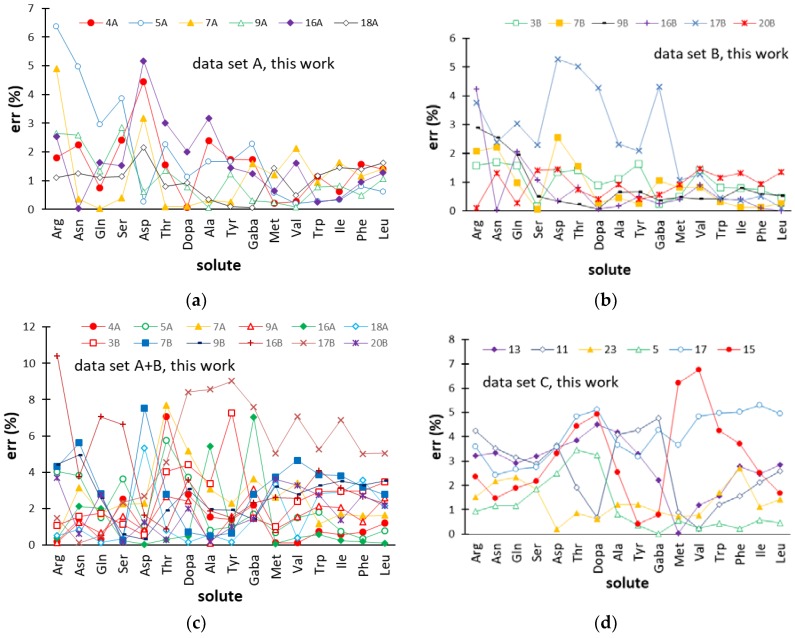
Percentage errors (err (%)) for the retention times of the amino acids provided by the ANN-based models in the external gradients (test set) of datasets A (**a**), B (**b**), A+B (**c**), and C (**d**). Abbreviations used for the amino acids are reported in Section 3.1. Gradient codes are specified in Table 1 (datasets A, B, and A+B) and Figure 1 (dataset C).

Figure 3 displays the trend of the relative (%) errors (err (%)) for the retention times of the 16 amino acids in the φ gradients and/or pH gradients of the test set. Therefore, these data quantify the ability of the ANN-based models to predict the retention of the amino acids in elution conditions external to those used in calibration, and give a measure of the applicability of this approach in optimization problems.

For most gradients of dataset A (φ gradients) and B (pH gradients), err (%) almost regularly decreased, passing from the less retained (Arg) to the most retained amino acid (Leu), seen from the left to the right of the plots displayed in Figure 3a,b. This arose from the fact that the absolute errors were homogeneously distributed over the target amino acids and, therefore, the relative errors were inversely related to the t_R_ value. Most of the predicted errors associated with the 16 amino acids in the external gradients of datasets A and B were smaller than 3%, while ANN modeling of datasets A+B (Figure 3c) and C (Figure 3d) provided slightly greater residuals, although generally below 5%. It can be noted that the retention times of most amino acids were less accurately predicted in the pH gradient 17B, when the data referring to pH gradients were modeled separately (dataset B) and when pH gradients and φ gradients were combined (dataset A+B). The moderately worse performance of the ANN model in this experimental condition can be due to the fact that the values of the two eluent descriptors (φ_i_ and pH_i_) of pH gradient 17B were the greatest within the respective variability ranges (Table 1) and, therefore, the network was called to extrapolate the response.

### 2.4. Comparison of the ANN-Based Models with Retention Models Based on the Solution of the Fundamental Equation of Gradient Elution

**Figure 4:**
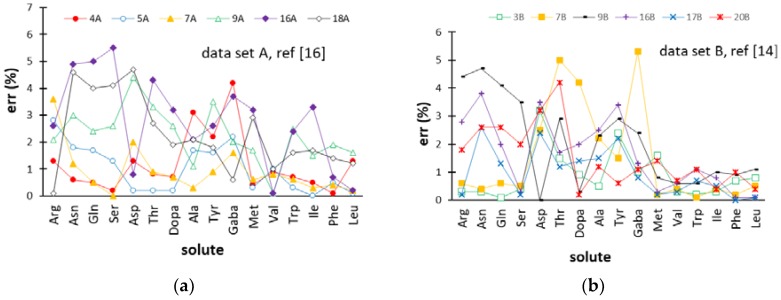
Percentage errors (err (%)) for the retention times of the amino acids provided by the retention models developed in References [14,16] for the external gradients (test set) of datasets A (**a**) and B (**b**). Abbreviations used for the amino acids are reported in Section 3.1. Gradient codes are specified in Table 1.

The error trends provided by the retention models based on the solution of the fundamental equation of gradient elution that Pappa-Louisi and co-workers applied to datasets A [16] and B [14] are displayed in Figure 4 for comparison purposes. With regards to dataset A, the ANN-based model gave a lower number of errors above 3% as compared with the retention model developed in Reference [16]. Concerning dataset B, it must be noted that the pH gradient retention data collected in the pH ranges of 2.8–10.7 and 3.2–9 (in Table 1, gradients 1B–10B and 11B–22B, respectively) were fitted by two separate models in Reference [14], while, in this work, all 22 elution conditions were modeled by the same network. Nevertheless, the comprehensive ANN-based model built here seems to give a better prediction of the retention times, whereby the number of errors above 2% was lower as compared with the results provided by the two separate retention models generated from the solution of the fundamental equation of gradient elution. Although t_R_ values of the most retained amino acids (Val, Trp, Ile, Phe, and Leu) were predicted by the two alternative approaches with a comparable accuracy (errors were close to 1% or lower), the behavior of the less retained solutes was better described by the ANN model.

The comparison of Figure 3c and Figure 4a,b reveals that the accuracy of prediction in the external φ gradients and pH gradients of dataset A+B was substantially equivalent to that provided by the solution of the fundamental equation of gradient elution. However, it should be remarked that a single ANN-based model was required to fit these data, while the data collected in pH gradients and φ gradients covering two different pH ranges were interpolated with three different retention models in References [14,16].

The ANN model describing retention under the application of double pH/φ gradients (dataset C) exhibited individual t_R_ errors in the external gradients that surpassed 5% only in a limited number of cases (Figure 3d). For this dataset, instead of the detailed trend of errors, not given in Reference [15], the mean errors provided by the retention model obtained from the solution of the fundamental equation of gradient elution could be considered for comparison. The mean and maximum errors reported for the model calibrated with all 27 gradients of dataset C were 2.9% and 18.9%, respectively. Moreover, the mean error associated with individual amino acids over the 27 gradients monotonically grew with the increase in retention time, from 1.5% (Arg) up to 6.5% (Leu) (Figure 5 of Reference [15]), revealing a poor modeling of the retention behavior of the most retained solutes. In the present work, the mean and maximum errors for the 17 gradients used to train the network were noticeably lower (1.0 and 4.2%, Table 2), and we observed a substantial independence of the training errors from the kind of amino acid. In Reference [15], the model initially developed using all the 27 gradients was recalibrated with 18 gradients and applied to the remaining nine gradients providing mean and maximum errors of 3.5 and 11.8%, respectively. In the present work, the network trained with 17 gradients gave lower mean and maximum errors both in internal validation (2.6 and 6.9%) and external prediction (2.5 and 6.8%). In summary, the ANN-based model, as compared with the retention models generated from the solution of the fundamental equation of gradient elution, provided a more accurate prediction of the retention times of the amino acids in double pH/φ gradients, as well as a more homogenous error distribution.

## 3. Methods

### 3.1. Retention Data

The data here analyzed were taken from three papers of Pappa-Louisi and co-workers [14,15,16] regarding the RP-HPLC retention of OPA derivatives of amino acids collected under the application of φ gradients, pH gradients, or combined pH/φ gradients. The mobile phases consisted of mixtures of aqueous phosphate buffer with a total ionic strength of 0.02 M and acetonitrile. In the first paper [16], 19 chromatographic runs were performed in different fixed eluent pHs (between 2.80 and 7.80), while the organic solvent volume fraction φ was linearly varied between 0.2 and 0.5 in different gradient durations (t_g_, ranging between 5 and 40 min). In the second paper [14], φ was kept fixed (at 0.25, 0.27, 0.3, or 0.35), and 22 different linear pH gradients were applied in the pH ranges of 2.8–10.7 or 3.2–9, where t_g_ was varied between 10 and 30 min. A third retention dataset (dataset C) was collected by Zisi et al. [15] under the application of a double organic solvent and pH gradient, in which both φ and pH were linearly changed from initial values (φ_i_ and pH_i_) to final values (φ_f_ and pH_f_). This consisted of 27 different runs performed at fixed values of φ_i_ (0.25) and pH_i_ (3.21), while pH_f_, φ_f_, and t_g_ were varied according to a three-level experimental design. The selected levels were 4.68, 5.86, and 7.86 for pH_f_; 0.35, 0.40, and 0.50 for φ_f_; and 10, 20, and 30 min for t_g_.

The amino acids analyzed in the above conditions were as follows: l-arginine (Arg), l-asparagine (Asn), l-glutamine (Gln), l-serine (Ser), l-aspartic acid (Asp), l-threonine (Thr), beta-(3,4-dihydroxyphenyl)-l-alanine (Dopa), l-alanine (Ala), l-tyrosine (Tyr), 4-aminobutyric acid (GABA), l-methionine (Met), l-valine (Val), l-tryptophan (Trp), l-isoleucine (Ile), l-phenylanine (Phe), and l-leucine (Leu). The amino acid l-glutamic acid (Glu), which was analyzed only in some experimental conditions, was not considered here. Apart from the different gradient profiles, all the retention data were collected with the same column, detector, and eluent flow rate. A 250 mm × 4.6 mm MZ-PerfectSil Target ODS-3HD analytical column with a 5-μm particle size kept at 25 °C was used, and the spectrofluorometric detector worked at 455 nm after excitation at 340 nm. Further experimental details can be found in the original papers [14,15,16].

### 3.2. Artificial Neural Network Modelling

A three-layer feed-forward ANN [30,31] was used in this work. The network consisted of one layer of input neurons, one output neuron, and an adjustable number of neurons in the hidden layer, fully connected to both the input and output neurons. Weights were associated to the connections, which modulated the information flowing from the input layer collecting the independent variables to the output neuron providing the network response. The weighted input variables entering each neuron of the hidden layer were summed and added to a bias value, and the result was transformed by a non-linear activation function, providing an output signal. The output neuron operated in a similar way on the weighted outputs of the hidden neurons producing the final response. A starting set of weights and biases, randomly generated, was sequentially updated in a learning (or training) procedure in which the network evaluated several input/output pairs (training set) to produce the best agreement between the target and computed responses. The optimized set of weights and biases, which represented a sort of memory of the learned network, could later be recalled, making predictions of the unknown response when the predictors were known. In this work, the network was trained by a quasi-Newton method [31], which incorporates second-order information about the error surface shape, ensuring faster convergence and a greater probability of avoiding local minima as compared to the classical error backpropagation learning algorithm. To avoid overfitting, the ANN performance during the learning step was monitored on unknown data samples (validation set), and the weight update was interrupted when the validation error started increasing. Minimization of the validation error was the criterion also adopted to select among alternative ANN models, differing in their network architecture, kind of activation function, kind of data scaling, and so on, the one with the best expected generalization ability. The real predictive performance of the final ANN-based model was finally evaluated on data samples (test set) external to both the training and validation sets. Software OpenNN [32] was used to perform ANN modeling.

## 4. Conclusions

In this paper, a three-layer artificial neural network was used to model the retention times of 16 amino acids under the separate or simultaneous application of linear organic modifier and pH gradients. We focused on the ANN’s capability to predict the retention data of the target solutes in external gradients, which is a useful response for optimization purposes. Using a “bit-string” representation of solutes allowed simultaneously modeling the retention behavior of all 16 amino acids with no explicit reference to their chemical structure or properties. It follows that the approach presented in this work can be transferred to chemical classes or heterogeneous groups of solutes different from those investigated. Moreover, the model generation did not require any assumption concerning the dependence of the retention factors on the eluent pH and composition, which is, by contrast, a prerequisite to attempt the solution of the fundamental equation of gradient elution. The predictive ability of the ANN-based models tested on external gradients was very good, whereby the mean errors for the retention times were 1.1% for φ gradients, 1.4% for pH gradients, and 2.5% for pH/φ gradients, and better than that provided by retention models based on the solution of the fundamental equation of gradient elution. In summary, ANN modeling seems a powerful and flexible regression tool to describe the effect of the experimental conditions in linear gradient elution on the retention of ionizable solutes and, in combination with experimental design, can be applied to optimize HPLC methods.

## Figures and Tables

**Table 1:** Descriptors of the linear φ gradients (dataset A) and pH gradients (dataset B).

Dataset	Gradient Code	Subset ^a^	pH_i_	ΔpH/t_g_	φ_i_	Δφ/t_g_
A	1A	train	2.8	0	0.20	0.06
	2A	val	2.8	0	0.20	0.03
	3A	train	2.8	0	0.20	0.015
	4A	test	3.3	0	0.20	0.03
	5A	test	3.3	0	0.20	0.02
	6A	train	3.3	0	0.20	0.015
	7A	test	3.3	0	0.20	0.01
	8A	train	3.82	0	0.20	0.06
	9A	test	3.82	0	0.20	0.018
	10A	train	3.82	0	0.20	0.012
	11A	train	4.2	0	0.20	0.03
	12A	train	4.2	0	0.20	0.015
	13A	val	4.2	0	0.20	0.01
	14A	val	5.85	0	0.20	0.015
	15A	train	5.85	0	0.20	0.01
	16A	test	5.85	0	0.20	0.0075
	17A	train	7.8	0	0.20	0.015
	18A	test	7.8	0	0.20	0.01
	19A	train	7.8	0	0.20	0.0075
B	1B	train	2.8	0.79	0.35	0
	2B	val	2.8	0.527	0.35	0
	3B	test	2.8	0.395	0.35	0
	4B	train	2.8	0.263	0.35	0
	5B	val	2.8	0.527	0.25	0
	6B	train	2.8	0.527	0.27	0
	7B	test	2.8	0.527	0.30	0
	8B	train	2.8	0.263	0.25	0
	9B	test	2.8	0.263	0.27	0
	10B	train	2.8	0.263	0.30	0
	11B	train	3.2	0.580	0.25	0
	12B	train	3.2	0.387	0.25	0
	13B	val	3.2	0.290	0.25	0
	14B	train	3.2	0.193	0.25	0
	15B	train	3.2	0.387	0.27	0
	16B	test	3.2	0.387	0.30	0
	17B	test	3.2	0.387	0.35	0
	18B	train	3.2	0.290	0.30	0
	19B	val	3.2	0.290	0.35	0
	20B	test	3.2	0.193	0.27	0
	21B	train	3.2	0.193	0.30	0
	22B	train	3.2	0.193	0.35	0

^a^ Training set (train), validation set (val), test set (test).

**Table 2:** Description of the ANN models developed on the retention datasets A, B, A+B, and C: architecture and learning duration of the optimal network, coefficients of determination in training (*R*^2^) and prediction (*Q*^2^) and respective standard errors (SEC and SEP), and the mean and maximum percentage errors (mean(%) and max(%)).

Data Set	Network Topology	Learning Epochs	Training				Validation				Test			
			*R* ^2^	SEC	mean(%)	max(%)	*Q* ^2^	SEP	mean(%)	max(%)	*Q* ^2^	SEP	mean(%)	max(%)
A	18-14-1^a^	251	0.9999	0.06	0.3	1.6	0.9906	0.37	1.6	7.4	0.9984	0.22	1.4	6.4
B	19-21-1	63	0.9980	0.46	0.7	4.1	0.9778	0.78	1.4	4.1	0.9949	0.48	1.1	5.3
A+B	20-23-1	286	0.9993	0.23	1.2	6.3	0.9939	0.65	3.3	12.6	0.9799	0.48	2.5	10.4
C	19-18-1	125	0.9994	0.22	1.0	4.2	0.9938	0.72	2.6	6.9	0.9958	0.59	2.5	6.8

^a^ Number of neurons in the input, hidden and output layer, respectively.
